# Understanding the relationship between eHealth literacy, self-efficacy, social support, and patient activation in breast cancer patients: a chain mediation analysis

**DOI:** 10.3389/fpubh.2026.1904185

**Published:** 2026-08-06

**Authors:** Lijia Hou, Jijun Wu, Jingru Zhou, Xuan Fu, Xiaoli Zhong

**Affiliations:** 1 Department of Nursing, Deyang People's Hospital, Deyang, Sichuan, China; 2 Department of Ophthalmology, Deyang People's Hospital, Deyang, Sichuan, China; 3 College of Nursing, Chengdu University of Traditional Chinese Medicine, Chengdu, Sichuan, China

**Keywords:** breast cancer, chain mediation, eHealth literacy, patient activation, self-efficacy, social support

## Abstract

**Background:**

Patient activation is essential for effective self-management among breast cancer patients, yet the mechanisms through which eHealth literacy contributes to it remain incompletely understood.

**Objective:**

This investigation sought to delineate the chain mediating roles of self-efficacy and social support linking eHealth literacy to patient activation among breast cancer populations.

**Methods:**

316 breast cancer patients were recruited into a cross-sectional survey framework. Each participant underwent an evaluation of their levels of patient activation, perceived social support, self-efficacy, and eHealth literacy. After controlling relevant factors, model 6 in PROCESS 4.1 was applied to construct a chain mediation model; the bootstrapping procedure was subsequently applied to verify the mediating pathways.

**Results:**

eHealth literacy showed a significant association with patient activation (*p* < 0.001) and demonstrated meaningful positive associations with self-efficacy and social support (*p* < 0.01). Chain mediation analysis revealed that social support and self-efficacy acted as sequential intermediaries transmitting the influence of eHealth literacy onto patient activation. The aggregate indirect effect accounted for 35.7%, with all pathway confidence intervals excluding zero.

**Conclusion:**

Among breast cancer patients, eHealth literacy was associated with patient activation through sequential mediation by social support and self-efficacy. These findings suggest that strategies to strengthen patients' eHealth literacy, mobilize social support, and build self-efficacy may hold promise for promoting active participation in health self-management, although the effectiveness of such interventions warrants confirmation in future longitudinal or intervention studies.

## 1 Introduction

Among women globally, breast cancer (BC) ranks as one of the most prevalent malignancies and contributes substantially to cancer-related morbidity and mortality. Data from the International Agency for Research on Cancer (IARC) show that 2022 saw approximately 2.3 million newly diagnosed BC cases and 670,000 cancer-related fatalities recorded globally, and the burden of illness will keep rising (1, 2). In 2022, there were about 357,200 new cases of BC in China, with a tendency toward younger individuals (3, 4). Advancements in therapeutic and diagnostic technologies have gradually repositioned BC as a long-term manageable condition (5); however, patients still face multiple psychological, physical, and social challenges throughout the course of their illness (6–8). As a result, a primary challenge in cancer care is identifying effective strategies to motivate BC patients to actively engage in managing their own health.

Patient activation (PA) refers to a patient's acquired knowledge, practical competencies, and self-assurance in navigating health management, alongside the drive and capacity to take measures to maintain health (9). It is an important indicator of patients' self-management. Previous studies have shown that highly activated cancer patients typically demonstrate better treatment adherence, stronger self-management abilities, and higher quality of life (10, 11), and are more proactive in participating in treatment decisions, symptom monitoring, and collaboration with the healthcare team (12). Studies (12, 13) consistently show that BC patients often have low-to-moderate levels of activation, which are regulated by a number of variables. Among these, health literacy has been identified as a factor associated with PA levels (14). Patients with higher health literacy, particularly those with BC, are often more adept at collecting and assessing information in medical consultations, which in turn motivates them to take a more active role in making treatment decisions (15).

eHealth literacy (eHL) denotes a person's proficiency in searching out, interpreting, and critically evaluating health information obtained through digital channels, employing this understanding to tackle health-related concerns (16). It has become increasingly indispensable in modern patient care. With the advancement of the Internet and mobile health technologies, patients are progressively migrating from traditional media to electronic platforms to obtain health-related knowledge. Studies suggest that individuals with higher proficiency in eHL can leverage online tools and mobile applications more efficiently (17). Research indicates that individuals with elevated eHL are better equipped to effectively and critically utilize digital resources, such as online platforms and mobile health applications, to enhance their understanding of self-care and gather information about diseases (17, 18), thereby demonstrating greater initiative and willingness to participate in their own health management. Nonetheless, the fundamental processes by which eHL additionally affects PA levels have yet to be fully investigated, and studies in this field remain limited, especially in the context of BC patients.

Social support enables people to experience feelings of being loved, cared for, respected and belonging to a social network (19). With the growing use of the internet, online patient forums and health communities have become important platforms through which BC patients can access such support. Greater eHL enables patients to more actively engage in these online exchanges, which in turn helps them build and sustain online social support networks more effectively (20). Beyond facilitating online social support in this way, greater social support has also been linked to broader health benefits among BC patients, including better quality of life, fewer symptoms of anxiety and depression, and a greater likelihood of adopting healthy behaviors (21, 22). Furthermore, social support from multiple sources has been proven to be a key source for enhancing patients' self-efficacy (23, 24). Specifically, emotional encouragement, informational support, and the sharing of experiences from healthcare providers, family members, and fellow patients can directly bolster patients' confidence in disease self-management, consequently elevating their self-efficacy levels (25). An improved sense of self-efficacy, in turn, promotes PA and encourages patients to embrace healthier behaviors; prior studies have shown that BC patients with elevated self-efficacy are more proficient at managing treatment-related challenges (26). Consequently, eHL and PA may be mediated in a consecutive chain by social support and self-efficacy.

Although previous studies have examined these relationships in isolation, the chain mediation effects involving eHL, social support, self-efficacy, and PA in BC survivors remain unverified. The present study seeks to establish a theoretical foundation for constructing targeted behavioral intervention frameworks by investigating how eHL influences PA and further exploring how social support and self-efficacy serially mediate this process among BC patients.

### 1.1 Theoretical framework

The theoretical underpinning of this study is grounded in the principles of Bandura's Social Cognitive Theory (SCT). This framework holds that behavior is not determined solely by internal traits or external circumstances, but rather by the ongoing dynamic interplay among personal cognition, behavior, and the environment (27). Self-efficacy constitutes a core construct of this framework, referring to one's perceived capacity and conviction to accomplish particular tasks successfully. It is an essential psychological process for forecasting and elucidating health behaviors (28). Additionally, the theory highlights the importance of vicarious experiences and observational learning in the development of positive health behaviors. It suggests that self-efficacy beliefs can be effectively reinforced by behavioral modeling, emotional support, and informational support from others (29).

Based on this model, this study proposes a serial mediation pathway to elucidate how health literacy translates into PA among BC patients: eHL → social support → self-efficacy → PA. Specifically, eHL represents the fundamental capacity to obtain, comprehend, and apply health information (individual level). BC patients with higher eHL can participate more actively in online health communities, gaining emotional and informational support through interaction, thereby expanding their social support networks (environmental level). Adequate social support reinforces patients' self-efficacy through emotional motivation and vicarious experiences, thereby driving their active participation in disease management and adherence to healthy behaviors, ultimately enhancing PA (behavioral level).

**Figure 1:**
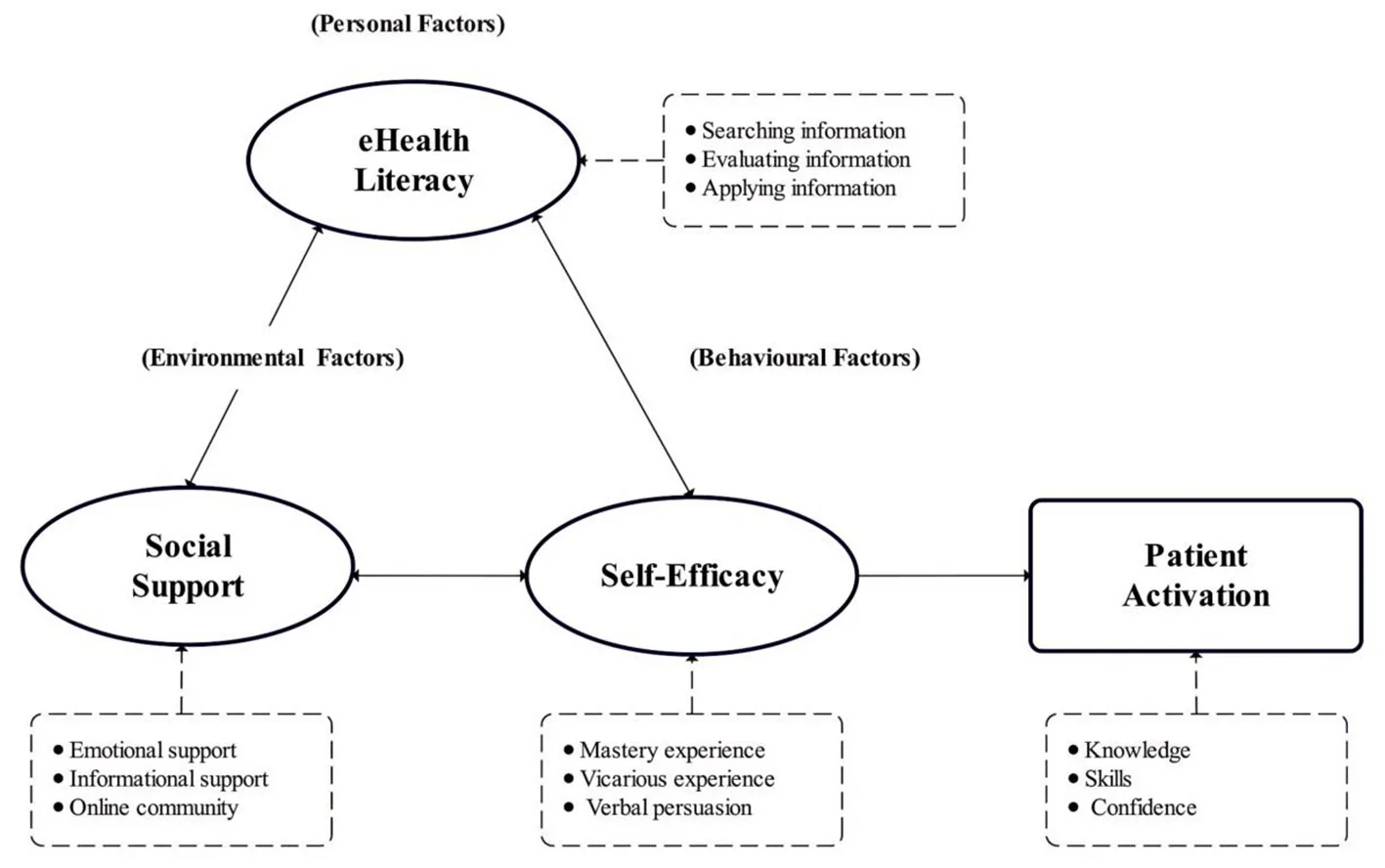
The conceptual framework.

In response to this, the research presents the subsequent theoretical hypotheses (refer to Figure 1):

Hypothesis 1: eHL positively and significantly predicts PA levels.

Hypothesis 2: Social support mediates the pathway through which eHL affects PA.

Hypothesis 3: Self-efficacy acts as a mediator between eHL and PA.

Hypothesis 4: Social support and self-efficacy together form a sequential mediation chain linking eHL to PA.

## 2 Methods

### 2.1 Study design and participants

A cross-sectional survey design was adopted. Clinical data were prospectively collected from both inpatients and outpatients attending a tertiary hospital's breast surgery and oncology unit in Deyang City between June and December 2025. Enrollment required participants to satisfy each of the following eligibility criteria: (1) aged 18 years or above; (2) BC diagnosis confirmed in accordance with the relevant clinical diagnostic guidelines (30) and having received pathological verification; (3) Mentally aware and skilled in communication, free from speech or language challenges, and able to complete the survey autonomously or with support. (4) Full awareness of their condition and willingness to participate voluntarily. Criteria for exclusion: (1) concurrent malignant tumors or severe life-threatening diseases; (2) mental disorders or cognitive impairment; (3) refusal to participate or poor questionnaire compliance; (4) terminally ill patients.

The estimation of the sample size was conducted utilizing the Monte Carlo power analysis technique, which was computed using the online software pwrSEM (https://schoemanna.shinyapps.io/mc_power_med/) (31). This power calculation was based on a chain mediation model, specified with power set at 0.80, a 95% confidence interval, and 5,000 bootstrap resamples. Results indicated that the statistical power for detecting mediation effects across all pathways, using the current sample size (*n* = 316), surpassed the critical threshold of 0.80, confirming the adequacy of the sample size.

### 2.2 Measures

#### 2.2.1 Demographic and clinical variables

A self-designed questionnaire captured sociodemographic profiles including residential location, age, marital status, occupational status, educational background, monthly household income per capita, medical payment method, and primary caregiving arrangements. Disease-related characteristics encompassed illness duration, tumor staging, treatment modality, the presence of distant metastases, and family history of breast cancer. Patterns of electronic device usage were additionally recorded.

#### 2.2.2 eHealth literacy

eHL was quantified using the Chinese-validated eHealth Literacy Scale (eHEALS), a validated eight-item instrument originally developed by Norman et al. (32) and subsequently localized for Chinese populations by Guo et al. (33). The instrument taps three competency domains: accessing and applying online health resources, critical appraisal of information, and evidence-informed decision-making. Each item is scored on a 5-point Likert continuum (1 = strongly disagree, 5 = strongly agree), producing total scores spanning 8 to 40; a score of 26 serves as the threshold distinguishing low from high eHL. Reliability was satisfactory, with a Cronbach's α of 0.837 in the current sample.

#### 2.2.3 Patient activation

Activation levels were captured via the localized Chinese version of the Patient Activation Measure (PAM), first developed by Hibbard et al. (34) and adapted into Chinese by Hong (35). Spanning 13 items across four subscales, responses follow a 5-point format (0 = not applicable, 1–4 = strongly disagree to strongly agree). Through an official algorithm, item scores are transformed into standardized values from 0 to 100, where elevated scores reflect greater levels of activation capacity. Four activation tiers are defined: level 1 ( ≤ 47.0), Level 2 (47.1–55.1), Level 3 (55.2–72.4), and Level 4 (≥72.5). Reliability in this sample was Cronbach's α = 0.791.

#### 2.2.4 Social support

Perceived social support was gauged by the Perceived Social Support Scale (PSSS), originally proposed by Zimet et al. (36), and subsequently revised by Jiang (37). Its 12 items are distributed equally among three support dimensions—family, friends, and significant others—each rated on a 7-point Likert continuum (1 = strongly disagree, 7 = strongly agree). Aggregate scores range from 12 to 84, where greater scores indicate more robust perceived support. The present sample returned a Cronbach's α of 0.884.

#### 2.2.5 Self-efficacy

Self-efficacy was indexed using the Chinese-language Breast Cancer Self-Efficacy Scale (BCSES), originally constructed by Champion et al. (38) and translated into Chinese by Yuan et al. (39). Intended to capture BC patients' perceived capability in managing symptoms and sustaining quality of life, the 11-item scale employs a 3-point Likert format, with total scores ranging from 11 to 55; higher scores reflect stronger self-efficacy beliefs. The scale demonstrated strong reliability (Cronbach's α = 0.880).

### 2.3 Ethical considerations

Ethical clearance for this study was granted by the Ethics Committee of Deyang People's Hospital (Approval No: 2025-04-047-K01). All enrolled participants provided written informed consent before data collection commenced. Each participant was fully briefed on the study's purpose, procedures, potential risks, and anticipated benefits. Enrollment was entirely voluntary; participants retained the right to discontinue at any point with no repercussions. Strict confidentiality was maintained over all personal data, which were accessed solely for research purposes.

### 2.4 Data collection

Eligible inpatients and outpatients were recruited by trained research personnel, who administered questionnaires individually in a designated private area after the participants' routine examinations or treatment sessions. Investigators provided neutral clarifications when items were unclear, refraining from influencing responses. Out of the 334 questionnaires distributed, those with a missing rate exceeding 20% or containing invalid entries (e.g., contradictory or repetitive patterns) were excluded, resulting in 316 usable responses, yielding a valid return rate of 94.61%.

### 2.5 Statistical analysis

Statistical procedures were carried out in SPSS 26.0 with the PROCESS macro (version 4.1), with a two-tailed significance threshold of *p* < 0.05. Continuous variables are expressed as the mean ± standard deviation; categorical variables are presented as frequencies and percentages. To screen for common method bias, the Harman single-factor test was conducted, and Pearson correlation analysis was used to characterize inter-variable associations. A chain mediation model was built using PROCESS macro Model 6, with path coefficients derived from four sequential regression analyses. Bootstrap resampling (5,000 iterations) was used to evaluate the mediating contributions of social support and self-efficacy within the eHL–PA relationship; effects were considered statistically significant when the 95% confidence interval excluded zero. All standardized effect sizes were computed from unstandardized coefficients.

## 3 Results

### 3.1 Common method bias test

Since the entire dataset relied on self-report measures, the potential for common method bias warranted examination. An unrotated principal component analysis was therefore performed across all scale items via the Harman single-factor test. The dominant common factor explained 23.20% of the total variance, falling well below the 40% benchmark. Furthermore, eight factors returned eigenvalues exceeding 1, collectively indicating no meaningful common method bias contamination in the present dataset.

### 3.2 General characteristics of participants

Among the 316 female BC patients enrolled, the mean age was 52.98 years. Regarding sociodemographic profiles, 34.2% resided in urban areas, 69.0% had a marital partner, and 63.6% held a high school qualification or above. Spouses (48.7%) and children (34.8%) constituted the predominant caregiver groups, and 64.8% of households reported a monthly per capita income of at least 3,000 yuan. Clinically, 71.2% presented with stage II–III tumors, 70.3% were free of distant metastases, and 78.9% had been diagnosed for over 6 months. Concerning digital health engagement, 34.8% had never accessed health-related websites or applications, while 51.3% accessed the internet on a daily basis (see Table 1).

**Table 1:** Demographic and clinical characteristics of participants (*N* = 316).

Characteristics	Categories	*n* (%)/M ±SD
Age (years)		52.98 ± 10.26
Place of residence	Urban	108 (34.2%)
	County town	62 (19.6%)
	Township	70 (22.2%)
	Rural	76 (24.1%)
Marital status	Married	218 (69.0%)
	Single	24 (7.6%)
	Divorced	39 (12.3%)
	Widowed	35 (11.1%)
Educational level	Primary school or below	48 (15.2%)
	Junior high school	67 (21.2%)
	High school/technical secondary school	111 (35.1%)
	College or above	90 (28.5%)
Monthly household income (per capita)	< ¥3,000	111 (35.1%)
	¥3,000–¥5,000	127 (40.2%)
	≥¥5,000	78 (24.7%)
Primary caregiver	Spouse	154 (48.7%)
	Children	110 (34.8%)
	Parents	20 (6.3%)
	Others	32 (10.1%)
Disease duration	≤ 6 months	67 (21.1%)
	6–12 months	79 (25.0%)
	13–24 months	88 (27.8%)
	>24 months	82 (25.9%)
Tumor stage (AJCC)	Stage I	65 (20.6%)
	Stage II	125 (39.6%)
	Stage III	100 (31.6%)
	Stage IV	26 (8.2%)
Distant metastasis	No	222 (70.3%)
	Yes	94 (29.7%)
Daily device time	< 1 h	121 (38.3%)
	1–3 h	114 (36.1%)
	>3 h	81 (25.6%)
Internet usage frequency	Rarely	50 (15.8%)
	Several times per week	104 (32.9%)
	Daily	162 (51.3%)
Use of health-related websites or apps	Never	110 (34.8%)
	Occasionally	117 (37.0%)
	Frequently	89 (28.2%)

M, mean; SD, standard deviation; AJCC, american joint committee on cancer.

### 3.3 Descriptive statistics and correlation analysis

Descriptive results revealed mean scores of 23.93 ± 5.91 for eHL, 46.05 ± 12.91 for PA, 47.76 ± 12.75 for social support, and 33.00 ± 7.93 for self-efficacy. Pairwise correlation analysis detected significant positive associations between eHL and PA (r = 0.535), self-efficacy (r = 0.405), and social support (r = 0.410), with all *p* < 0.01. Social support was likewise positively associated with both self-efficacy (r = 0.363) and PA (r = 0.480) (*p* < 0.01), and self-efficacy showed a significant positive link with PA (r = 0.440, *p* < 0.01). These inter-variable correlations confirmed that the prerequisite conditions for mediation analysis were met. Additionally, eHL was inversely associated with age (r = −0.222, *p* < 0.01), whereas eHL showed positive correlations with educational attainment, duration of electronic device usage, frequency of internet engagement, and utilization of health-related websites or applications (r = 0.142–0.216, *p* < 0.05). Accordingly, these covariates were incorporated into all subsequent regression models (refer to Table 2).

**Table 2:** Descriptive statistics and correlations among study variables (*n* = 316).

Variable	Mean	SD	Range	1	2	3	4	5	6	7	8	9
1 Age	52.98	10.19	30~78	1								
2 Educational attainment	—	—	—	−0.430^**^	1							
3 Daily device time	—	—	—	−0.353^**^	0.212^**^	1						
4 Internet usage frequency	—	—	—	−0.310^**^	0.181^**^	0.077	1					
5 Use of health-related websites or apps	—	—	—	−0.144^*^	0.127^*^	0.068	0.441^**^	1				
6 eHealth literacy	23.93	5.91	8~39	−0.222^**^	0.196^**^	0.142^*^	0.216^**^	0.201^**^	1			
7 Social support	47.76	12.75	13~78	−0.133^*^	0.066	−0.103	0.067	−0.032	0.410^**^	1		
8 Self-efficacy	33.00	7.93	13~55	−0.053	0.134^*^	−0.017	0.071	−0.050	0.405^**^	0.363^**^	1	
9 Patient activation	46.05	12.91	17.9~90.7	−0.038	0.100	0.061	0.011	−0.023	0.535^**^	0.480^**^	0.440^**^	1

Variables 2–5 are categorical and were dummy–coded for correlation analysis. ^*^p < 0.05. ^**^p < 0.01.

### 3.4 Chain mediation analysis

With the aim of clarifying the serial mediating roles of social support and self-efficacy in the eHL–PA pathway, Model 6 from PROCESS 4.1 was employed, with age, educational attainment, daily electronic device usage, internet engagement frequency, and health-related website or app utilization entered as covariates.

**Figure 2:**
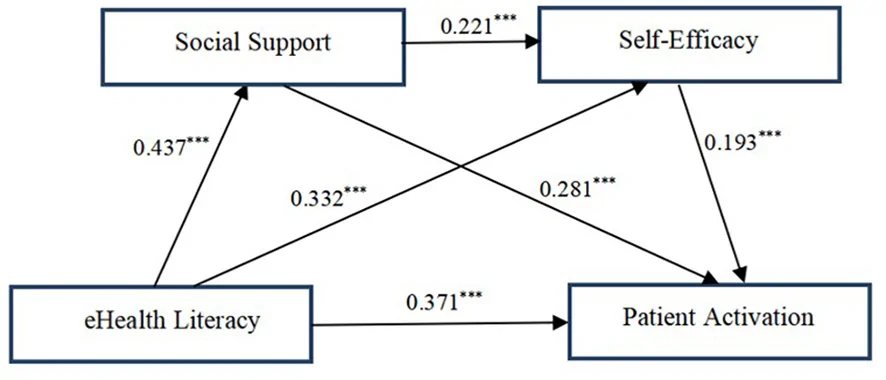
Diagram of the mediating model of social support and self-efficacy between ehealth literacy and patient activation.

Pathway-specific regression coefficients are detailed in Table 3 and illustrated in Figure 2. The findings affirmed the primary association, whereby eHL was significantly linked to PA (β = 0.577, *p* < 0.001). thereby supporting Hypothesis 1. In the presence of mediators, eHL demonstrated robust positive associations with both social support (β = 0.437, *p* < 0.001) and self-efficacy (β = 0.332, *p* < 0.001). Furthermore, social support proved a significant positive determinant of both self-efficacy (β = 0.221, p < 0.001) and PA (β = 0.281, *p* < 0.001). Additionally, self-efficacy functioned as a robust predictor of PA (β = 0.193, *p* < 0.001). The direct relationship between eHL and PA retained statistical significance following mediation adjustment (β = 0.371, *p* < 0.001).

**Table 3:** Path regression coefficients of the chain mediation model (*n* = 316).

Regression equation		Fitting index			Significance	
Outcome variable	Predictor variable	*R*	*R* ^2^	*F*	β	*t*
Patient activation		0.559	0.313	23.422^***^		
	Age				0.008	1.431
	Education level				0.039	0.804
	Daily device time				0.013	0.208
	Internet usage frequency				−0.064	−0.854
	Use of health-related websites or apps				−0.142	−2.123^*^
	eHealth literacy				0.577	11.596^***^
Social support		0.469	0.220	14.550^***^		
	Age				−0.013	−2.163^*^
	Education level				−0.018	−0.344
	Daily device time				−0.252	−3.693^***^
	Internet usage frequency				0.009	0.108
	Use of health-related websites or apps				−0.158	−2.221^*^
	eHealth literacy				0.437	8.249^***^
Self-efficacy		0.485	0.235	13.517^***^		
	Age				0.008	1.343
	Education level				0.095	1.855
	Daily device time				−0.037	−0.531
	Internet usage frequency				0.070	0.894
	Use of health–related websites or apps				−0.167	−2.341^*^
	eHealth literacy				0.332	5.716^***^
Social support				0.221	3.913^***^
Patient activation		0.650	0.423	28.098^***^		
	Age				0.011	2.045^*^
	Education level				0.026	0.588
	Daily device time				0.102	1.692
	Internet usage frequency				−0.080	−1.166
	Use of health-related websites or apps				−0.059	−0.937
	eHealth literacy				0.371	6.991^***^
Social support				0.281	5.579^***^
Self-efficacy				0.193	3.897^***^

^*^p < 0.05, ^**^p < 0.01, ^***^p < 0.001, β = unstandardized regression coefficient.

As displayed in Table 4, eHL's total effect on PA amounted to 0.577 [95% CI = (0.479, 0.674)]. The chained mediators contributed 35.7% to this total, resulting in an aggregate indirect effect of 0.206 [95% CI = (0.139, 0.278)]. This was channeled through three routes: the first (eHL → social support → PA) contributed 0.123 [59.7% of indirect; 95% CI = (0.076, 0.179)], confirming the mediating role of social support between eHL and PA and thereby supporting Hypothesis 2; the second (eHL → self-efficacy → PA) added 0.064 (31.1% of indirect; 95% CI = [0.029, 0.107]), verifying the mediating role of self-efficacy between eHL and PA and thereby supporting Hypothesis 3; and the full serial path (eHL → social support → self-efficacy → PA) added 0.019 [9.2% of indirect; 95% CI = (0.007, 0.036)], confirming the sequential (chain) mediating role of social support and self-efficacy between eHL and PA and thereby supporting Hypothesis 4. The direct effect constituted the remaining 0.371 (64.3%). As all indirect effects were based on non-zero Bootstrap 95% confidence intervals, they were all statistically significant, and taken together, these results confirm that all four hypotheses (H1–H4) were supported.

**Table 4:** Bootstrap analysis of chain mediation effects.

Path	Effect	SE	95% CI	Relative effect
Total effect	0.577	0.05	[0.479, 0.674]	—
Direct effect	0.371	0.053	[0.267, 0.476]	—
Total indirect effect	0.206	0.035	[0.139, 0.278]	100.00%
Ind1: X → M1 → Y	0.123	0.026	[0.076, 0.179]	59.70%
Ind2: X → M2 → Y	0.064	0.02	[0.029, 0.107]	31.10%
Ind3: X → M1 → M2 → Y	0.019	0.008	[0.007, 0.036]	9.20%

Boot SE, Standard error of the indirect effect; X, eHealth Literacy; M1, Social Support; M2, Self-Efficacy; Y, Patient Activation; CI, confidence interval.

## 4 Discussion

According to the results, the total eHL score for BC patients was 23.93 ± 5.91, indicating an inadequate level of digital proficiency. This is consistent with earlier investigations by Shan (40) and Fu (41), which highlight the necessity to enhance digital literacy, as patients generally demonstrate insufficient skills in retrieving and applying digital health information. In parallel, this study revealed a mean PA score of 46.05 ± 12.91, categorizing them at Level 1. This value is markedly inferior to scores reported in early-stage Dutch BC patients (42) and relevant Chinese cohorts (12). These data suggest that the overall engagement in health management was subpar, revealing deficiencies in self-management consciousness and the ability to participate in medical decisions. Several factors may account for this phenomenon: the elevated mean age of the cohort, a paucity of active decision-making attitudes, geographic barriers limiting access to health education for rural residents, and the influence of traditional medical beliefs embedded in Chinese medical culture, which often predisposes patients toward passive compliance with medical advice (42, 43).

This study found that eHL was directly associated with BC patients' PA levels, consistent with earlier research findings (44, 45). The underlying mechanism could be that eHL, as a crucial tool for people to access, comprehend, and use health information, may be associated with a broader knowledge base about health and a greater capacity to filter and make decisions when confronted with complex medical information, which in turn may relate to greater confidence and initiative in engaging with health management (46). Additionally, individuals with elevated eHL demonstrate greater skills in communicating and collaborating with healthcare teams through digital health platforms, which fosters favorable doctor-patient collaborative relationships and further encourages self-management behaviors (47). Consequently, in clinical practice, healthcare professionals should prioritize improving patients' eHL and regard it as a pivotal intervention strategy for elevating the PA levels of BC patients.

The results confirm that social support serves as an intermediary mechanism linking eHL to PA. According to research, individuals with low eHL have trouble accessing, comprehending, and digesting online health information, which makes it difficult for them to get the care they need (48). Conversely, individuals with elevated eHL tend to be more skilled at proactively pursuing and utilizing social support resources through digital platforms, which may be associated with greater access to both emotional and informational assistance (49). Moreover, individuals with higher eHL tend to report more satisfactory social support (50). In order to translate the capacity-building advantages of eHL into real health management behaviors and, ultimately, raise the PA level of BC patients, social support can successfully close gaps in patients' access to information, emotional control, and decision-making involvement.

The present findings further revealed that self-efficacy functions as an intervening variable within the eHL–PA relationship. This outcome is consistent with previous studies on chronic illnesses, which suggest that self-efficacy serves as a bridge between eHL and self-management behaviors, and that the association between eHL and self-management appears stronger when self-efficacy is taken into account (51). Consistent with SCT, self-efficacy is viewed as a situational cognitive judgment that individuals develop progressively through ongoing interactions with their environment, rather than as a fixed personality trait (28). BC patients' self-efficacy beliefs are gradually strengthened through the accumulation of successful management experiences; this improvement increases their ability and eagerness to participate actively in the processes of diagnosis and treatment (26). Additionally, the present study revealed that BC patients who possess greater self-efficacy exhibit increased confidence in their ability to understand and utilize health information, even when encountering intricate materials. Therefore, by offering positive feedback and sharing success stories, healthcare providers can increase patients' self-efficacy while also enhancing their eHL, thereby promoting their PA.

This study also revealed that by enhancing BC patients' social support and self-efficacy, eHL can have a positive impact on their PA level. Specifically, greater perceived social support was associated with higher self-efficacy among BC patients, which in turn was associated with higher levels of PA. This could be the fundamental mechanism: First, patients with higher eHL can expand their social support networks by utilizing online platforms, patient communities, and online medical resources more effectively (20). Second, a robust social support system not only improves patients' psychological coping skills and mitigates the psychological stress induced by the illness but it also appears to be associated with more favorable health-related behaviors and higher self-efficacy in managing their condition (24). Last but not least, self-efficacy is a crucial psychological motivator that can help patients transition from passively receiving treatment to actively managing their health, demonstrating higher PA levels (26). Notably, this integrated pathway aligns with evidence from cardiac populations, where Liu et al. reported that social support and self-efficacy serially mediated the association between health literacy and self-management behaviors (52). Therefore, it might be feasible to successfully encourage active patient participation in treatment and enhance overall health outcomes by harnessing the joint contributions of eHL, social support, and self-efficacy.

## 5 Limitations

The present study has certain inherent limitations. Firstly, the use of a cross-sectional approach prevents any determination of causality or temporal ordering among the variables; therefore, prospective longitudinal investigations are warranted to validate these associations. Furthermore, the exclusive reliance on self-report questionnaires for all measurements may introduce subjectivity and potential response bias. Strengthening the validity and comprehensiveness of future findings would require incorporating objective measures or adopting mixed-method approaches. In addition, the definition of PA adopted in this study incorporates “acquired knowledge” as one of its components, which conceptually overlaps with the information-comprehension ability described in the definition of eHL. This overlap may introduce shared variance, potentially contributing to the relatively high correlation observed between eHL and PA. Future studies could examine the discriminant validity between the two constructs by refining scale items or applying confirmatory factor analysis. Additionally, the geographical representativeness of the sample is limited, as data collection was confined to healthcare institutions within a single region. Broader sampling across hospitals of different tier levels and geographic areas would enhance the generalizability of these findings. Finally, the specified chain mediation model accounts for only a portion of the potential indirect pathways, suggesting that other mediators (e.g., health attitudes, treatment adherence, and doctor-patient communication) beyond social support and self-efficacy may exist. Further investigation is necessary to refine and expand the theoretical model.

## 6 Conclusion

In conclusion, BC patients generally exhibit low PA levels and inadequate eHL. Beyond its direct association with PA levels, eHL was also indirectly associated with PA via a sequential chain mediation pathway through social support and self-efficacy. These findings lay a theoretical groundwork for upcoming studies and strategies concerning PA among BC patients. Additionally, toward the goal of optimizing health outcomes and encouraging greater patient participation in self-management, it is recommended that healthcare providers develop interventions aimed at enhancing electronic health literacy, broadening social support networks, and boosting self-efficacy.

## Data Availability

The raw data supporting the conclusions of this article will be made available by the authors, without undue reservation.
